# Supplementary material for: Volatile Profiling of Pleurotus eryngii and Pleurotus ostreatus Mushrooms Cultivated on Agricultural and Agro-Industrial By-Products
Source: Foods. 2021 Jun 4;10(6):1287. doi: 10.3390/foods10061287 (PMC8226524; doi:10.3390/foods10061287)

SUPPLEMENTARY MATERIAL

Volatile Profiling of *Pleurotus Eryngii* and *P. Ostreatus* Cultivated on Agricultural and Agro Industrial By-Products

Dimitra Tagkouli [1], Georgios Bekiaris [2], Stella Pantazi [1], Maria Eleni Anastasopoulou [1], Georgios Koutrotsios [2], Athanasios Mallouchos [3], Georgios I. Zervakis [2] and Nick Kalogeropoulos [1,*]

[1] Harokopio University of Athens, Department of Dietetics-Nutrition, School of Health Science and Education, El. Venizelou 70, Kallithea, 176 76 Athens, Greece; dtagkoul@hua.gr (DT); stellapant15@gmail.com (SP); maria_anastaso@outlook.com (MEA)

[2] Agricultural University of Athens, Laboratory of General and Agricultural Microbiology, Iera Odos 75, 11855 Athens, Greece; giorgosbekiaris@yahoo.gr (GB); georgioskoutrotsios@gmail.com (GK); zervakis@aua.gr (GIZ)

[3] Agricultural University of Athens, Department of Food Science and Human Nutrition, Iera Odos 75, 11855 Athens, Greece; amallouchos@aua.gr (AM)

Table S1. Volatile compounds (μg/g f.w. equivalents of 4-methyl-2-pentanol) in *Pleurotus eryngii* (strains CS1, CS2, CS3 and LGAM106) mushrooms cultivated on different substrates (WS, GM and OL).

Class of compound	Compound	Rt (min)	RI	*P. eryngii*											
				CS1			CS2			CS3			LGAM106		
				WS	GM	OL	WS	GM	OL	WS	GM	OL	WS	GM	OL
Alkane	1,3-octadiene	3.75	966	n.d.	n.d.	0.68	n.d.	0.78	n.d.	1.30	n.d.	n.d.	1.91	n.d.	2.43
Aromatic	toluene	4.58	1048	18.27	18.04	25.88	17.29	15.84	9.78	15.45	13.86	15.71	21.37	8.87	20.02
Aldehyde	n-hexanal	4.98	1087	66.94	54.75	29.68	26.38	27.57	21.38	55.4	63.1	20.33	50.42	37.22	15.14
Alkane	undecane	5.11	1100	8.98	16.7	17.63	16.45	12.37	20.81	15.53	20.18	16.89	7.93	10.37	17.99
Aromatic	ethylbenzene	5.40	1129	0.92	1.28	1.19	0.43	0.63	n.d.	1.19	0.9	1.17	0.78	n.d.	n.d.
Aldehyde	heptanal	6.00	1189	6.36	2.88	n.d.	n.d.	n.d.	n.d.	n.d.	n.d.	n.d.	n.d.	n.d.	n.d.
Alkane	dodecane	6.11	1200	n.d.	n.d.	n.d.	0.36	0.2	0.37	n.d.	n.d.	n.d.	n.d.	1.14	n.d.
Terpene	limonene	6.16	1205	n.d.	0.45	0.34	n.d.	n.d.	n.d.	n.d.	n.d.	n.d.	n.d.	0.07	n.d.
Aldehyde	2-hexenal	6.37	1224	0.97	0.38	n.d.	n.d.	0.13	n.d.	0.1	0.42	n.d.	0.55	n.d.	n.d.
Furan	2-pentylfuran	6.47	1233	0.56	0.53	n.d.	0.30	n.d.	n.d.	0.17	0.33	n.d.	0.49	n.d.	n.d.

Class of compound	Compound	Rt (min)	RI	*P. eryngii*											
				CS1			CS2			CS3			LGAM106		
				WS	GM	OL	WS	GM	OL	WS	GM	OL	WS	GM	OL
Ketone	3-octanone	6.75	1259	59.62	72.5	68.07	37.23	40.2	22.1	43.09	51.2	49.41	70.17	38.89	59.59
Ketone	2-octanone	7.09	1291	3.75	3.44	n.d.	1.73	1.74	0.24	1.95	2.27	0.58	2.79	n.d.	n.d.
Aldehyde	n-octanal	7.14	1295	17.8	15.85	27.5	2.57	3.09	2.85	16.29	14.51	28.99	29.78	10.63	42.26
Alkane	tridecane	7.19	1300	n.d.	n.d.	n.d.	n.d.	n.d.	n.d.	n.d.	11.46	n.d.	n.d.	3.64	n.d.
Ketone	1-octen-3-one	7.29	1308	12.19	11.06	11.23	4.13	4.41	3.3	10.41	11.47	9.79	14.22	8.23	12.82
Ketone	2,3-octanedione	7.50	1324	6.96	5.3	2.91	2.44	1.48	3.32	4.7	3.84	2.95	4.21	5.00	1.65
Aldehyde	2-heptenal	7.60	1332	25.18	27.11	9.56	9.95	7.49	5.09	18.51	20.2	8.7	19.5	13.66	7.19
Ketone	6-methyl-5-hepten-2-one	7.72	1341	0.63	n.d.	n.d.	0.38	0.27	n.d.	n.d.	0.4	0.45	n.d.	n.d.	n.d.
Alcohol	1-hexanol	7.89	1355	1.48	1.29	0.38	3.14	2.1	2.51	0.97	2.4	n.d.	9.18	4.13	4.05
Alcohol	3-octanol	8.42	1396	26.59	27.7	16.71	9.46	15.69	5.6	24.16	17.99	7.96	14.01	n.d.	12.36
Alkane	tetradecane	8.47	1400	n.d.	n.d.	n.d.	n.d.	n.d.	n.d.	n.d.	15.97	n.d.	n.d.	8.92	n.d.
Aldehyde	nonanal	8.48	1401	4.41	3.6	2.44	0.99	1.4	1.41	3.31	2.31	4.15	3.19	n.d.	3.52
Ketone	3-octen-2-one	8.71	1416	2.78	5.27	1.01	2.45	1.85	1.5	3.27	3.96	1.33	3.32	2.36	n.d.
Alkane	3-ethyl-2-methyl-1,3-hexadiene	8.91	1429	1.10	n.d.	n.d.	n.d.	n.d.	n.d.	1.29	0.99	n.d.	n.d.	n.d.	n.d.
Aldehyde	2-octenal	9.04	1437	36.5	33.04	46.41	16.22	9.99	7.54	36.83	32.67	25.68	59.57	35.88	34.45
Alcohol	1-octen-3-ol	9.27	1452	576.09	555.8	870.52	335.79	423.1	308.31	477.19	570.84	771.37	826.8	708.25	1039.54
Alcohol	2-ethylhexanol	9.89	1494	2.22	1.53	1.6	1.86	1.37	1.75	1.86	1.24	1.36	1.18	0.34	1.25
Alkane	pentadecane	9.99	1500	n.d.	n.d.	n.d.	n.d.	n.d.	n.d.	n.d.	10.22	n.d.	n.d.	8.10	n.d.
Aldehyde	benzaldehyde	10.56	1533	23.31	21.76	13.08	8.14	4.71	4.77	17.28	18.99	6.57	41.78	29.9	22.3
Alcohol	1-octanol	11.06	1562	1.62	1.34	2.5	3.84	4.4	4.36	1.51	2.17	4.1	35.47	38.59	78.54
Alkane	hexadecane	11.72	1600	n.d.	n.d.	n.d.	n.d.	n.d.	n.d.	n.d.	7.13	13.3	n.d.	3.57	n.d.
Ketone	2-undecanone	11.81	1605	0.68	0.54	n.d.	0.23	n.d.	n.d.	0.48	0.57	n.d.	0.49	n.d.	n.d.

Class of compound	Compound	Rt (min)	RI	*P. eryngii*											
				CS1			CS2			CS3			LGAM106		
				WS	GM	OL	WS	GM	OL	WS	GM	OL	WS	GM	OL
Alcohol	2-octen-1-ol	12.11	1621	3.9	3.58	5.14	2.41	3.09	4.4	3.46	4.11	9.88	17.99	47.05	41.94
Aldehyde	phenylacetaldehyde	12.59	1646	2.98	1.72	n.d.	1.29	1.17	n.d.	1.45	1.71	n.d.	n.d.	n.d.	n.d.
Ketone	acetophenone	12.82	1658	1.8	1.12	n.d.	n.d.	0.44	n.d.	1.24	1.17	n.d.	n.d.	n.d.	n.d.
Terpene	pristane	12.98	1667	n.d.	n.d.	n.d.	n.d.	n.d.	n.d.	n.d.	8.21	15.44	n.d.	3.92	n.d.
Alkane	heptadecane	13.60	1700	n.d.	n.d.	n.d.	n.d.	n.d.	n.d.	n.d.	4.86	11.28	n.d.	3.19	n.d.
Aldehyde	2,4-nonadienal	13.74	1707	10.58	7.44	2.04	3.22	2.79	1.67	6.61	5.71	2.88	6.37	4.49	1.36
Aldehyde	undecenal	14.68	1756	1.25	n.d.	n.d.	n.d.	n.d.	n.d.	1.49	1.83	2.48	n.d.	n.d.	n.d.
Aldehyde	3-dodecen-1-al	14.68	1756	n.d.	n.d.	4.45	n.d.	n.d.	n.d.	1.11	n.d.	n.d.	2.73	n.d.	n.d.
Aldehyde	2,4 decadienal	14.92	1768	1.25	0.9	0.81	0.94	n.d.	0.42	0.74	1.13	0.56	0.88	n.d.	n.d.
Terpene	phytane	15.03	1774	n.d.	n.d.	n.d.	n.d.	n.d.	n.d.	n.d.	5.51	11.2	n.d.	n.d.	n.d.
Alkane	octadecane	15.53	1800	n.d.	0.65	n.d.	n.d.	n.d.	n.d.	n.d.	2.93	9.74	n.d.	4.82	n.d.
FAME	methyl laurate	15.65	1806	n.d.	0.7	1.47	0.27	0.33	0.34	0.84	0.72	0.77	n.d.	0.28	1.22
Aldehyde	2,4 decadienal	15.81	1814	2.51	1.49	1.34	0.57	n.d.	0.64	1.49	1.12	1.09	1.65	n.d.	0.99
Alkane	n-nonadecane	17.49	1900	n.d.	0.42	n.d.	n.d.	n.d.	n.d.	n.d.	2.13	6.79	n.d.	4.83	n.d.
Alcohol	phenylethyl alcohol	17.79	1916	n.d.	n.d.	n.d.	1.79	0.61	n.d.	n.d.	0.53	n.d.	n.d.	n.d.	n.d.
Alcohol	1-dodecanol	18.89	1973	n.d.	n.d.	1.72	n.d.	0.42	n.d.	n.d.	n.d.	n.d.	n.d.	n.d.	n.d.
Alkane	eicosane	19.41	2000	n.d.	n.d.	n.d.	n.d.	n.d.	n.d.	n.d.	1.11	3.68	n.d.	2.75	n.d.
FAME	methyl myristate	19.64	2012	2.16	2.82	4.1	0.83	1.75	1.55	2.2	4.07	3.97	1.61	5.27	5.3
Alkane	heneicosane	21.30	2100	n.d.	n.d.	n.d.	n.d.	n.d.	n.d.	n.d.	n.d.	1.21	n.d.	n.d.	n.d.
FAME	methyl pentadecanoate	21.58	2116	5.06	4.56	6.17	1.74	2.23	1.6	4.43	6.98	7.57	2.76	9.57	5.42
FAME	methyl palmitate	23.46	2221	20.51	24.13	31.69	11.74	12.39	9.37	19.12	33.55	62.38	30.61	46.56	75.27
FAME	methyl stearate	27.02	2413	n.d.	1.07	1.22	n.d.	n.d.	n.d.	0.6	1.57	2.09	n.d.	n.d.	2.37

Class of compound	Compound	Rt (min)	RI	*P. eryngii*											
				CS1			CS2			CS3			LGAM106		
				WS	GM	OL	WS	GM	OL	WS	GM	OL	WS	GM	OL
FAME	methyl oleate	27.36	2440	13.45	16.15	25.51	9.52	10.05	6.44	12.43	23.34	45.16	11.68	41.57	48.01
FAME	methyl linoleate	28.15	2503	14.89	13.07	17.12	5.84	5.15		8.3	11.08	21.09	22.47	8.9	27.7

*Results are averages of three replicates; n.d.: not detected; CS: commercial strain; LGAM: Laboratory of General and Agricultural Microbiology (Agricultural University of Athens, Greece); FAME: fatty acid methyl ester; WS, wheat straw; GM, wheat straw with grape marc (1:1 w/w); OL, olive leaves with olive mill wastes (3:1 w/w ratio)

Table S2. Volatile compounds (μg/g f.w. equivalents of 4-methyl-2-pentanol) in *Pleurotus ostreatus* (strains CS4, CS5, CS6 and LGAM3002) mushrooms cultivated on different substrates (WS, GM and OL).

Class of compound	Compound	Rt (min)	RI	*P. ostreatus*											
				CS4			CS5			CS6			LGAM3002		
				WS	GM	OL	WS	GM	OL	WS	GM	OL	WS	GM	OL
Alkane	1,3-octadiene	3.75	966	1.63	3.43	n.d.	2.45	n.d.	n.d.	2.98	3.98	n.d.	1.45	3.58	2.02
Aromatic	toluene	4.58	1048	65.3	65.3	59.51	194.02	123.51	98.86	114.05	110.35	90.37	99.15	126.1	104.62
Aldehyde	n-hexanal	4.98	1087	25.35	32.77	8.02	31.24	43.5	19.84	38.92	8.33	20.61	25.44	19.67	26.44
Alkane	undecane	5.11	1100	12.3	7.41	9.91	27.38	26.29	26.38	24.68	7.93	21.94	8.99	21.12	21.98
Aldehyde	heptanal	6.00	1189	n.d.	2.10	n.d.	n.d.	n.d.	n.d.	0.45	n.d.	n.d.	n.d.	n.d.	n.d.
Alkane	dodecane	6.11	1200	0.28	n.d.	n.d.	1.18	n.d.	n.d.	n.d.	n.d.	0.46	n.d.	1.47	n.d.
Terpene	limonene	6.16	1205	0.09	n.d.	n.d.	3.75	2.22	1.79	0.09	n.d.	0.19	2.33	4.01	0.44
Aldehyde	2-hexenal	6.37	1224	0.28	0.29	n.d.	n.d.	n.d.	n.d.	n.d.	n.d.	0.47	0.46	0.32	n.d.
Furan	2-pentylfuran	6.47	1233	n.d.	0.94	n.d.	0.84	n.d.	0.62	n.d.	n.d.	0.64	0.47	0.56	n.d.
Ketone	3-octanone	6.75	1259	118.97	142.24	61.77	371.98	328.25	315.09	234.05	283.02	284.55	211.16	284.17	363.08
Aldehyde	n-octanal	7.14	1295	5.97	25.02	n.d.	9.76	8.33	10.92	7.86	18.24	6.23	14.18	8.65	7.26
Ketone	1-octen-3-one	7.29	1308	9.67	24.09	5.83	16.58	18.88	14.14	8.21	9.91	11.43	15.84	22.56	22.00
Ketone	2,3-octanedione	7.50	1324	1.52	1.83	0.31	2.24	4.14	1.91	2.4	1.1	1.5	1.93	1.74	0.81
Aldehyde	2-heptenal	7.60	1332	16.52	18.46	3.5	12.17	11.77	18.2	6.17	9.23	10.92	11.26	14.55	9.18
Alcohol	1-hexanol	7.89	1355	1.02	2.86	10.99	1.36	0.75	n.d.	n.d.	n.d.	1.22	1.00	2.55	1.41
Alcohol	3-octanol	8.42	1396	69.79	253.29	83.07	600.13	777.2	754.7	803.14	830.78	564.79	331.79	681.6	526.7

Class of compound	Compound	Rt (min)	RI	*P. ostreatus*											
				CS4			CS5			CS6			LGAM3002		
				WS	GM	OL	WS	GM	OL	WS	GM	OL	WS	GM	OL
Alkane	3-ethyl-2-methyl-1,3-hexadiene	8.91	1429	1.01	0.96	n.d.	n.d.	n.d.	1.45	n.d.	n.d.	1.58	0.62	1.42	n.d.
Aldehyde	2-octenal	9.04	1437	14.01	40.8	4.21	39.51	41.45	24.7	11.09	14.94	17.5	22.92	26.76	7.05
Alcohol	1-octen-3-ol	9.27	1452	424.98	738	151.73	846.2	868.14	639.62	801.75	799.19	465.19	699.6	784.3	589.58
Alcohol	2-ethylhexanol	9.89	1494	1.03	n.d.	2.07	n.d.	1.92	1.67	2.48	1.9	1.75	1.66	1.43	1.69
Aldehyde	benzaldehyde	10.56	1533	2.01	7.27	2.32	9.00	12.3	5.81	4.84	5.12	4.2	6.47	5.06	2.51
Alcohol	1-octanol	11.06	1562	2.64	7.36	2.53	8.71	12.49	4.87	4.9	4.47	3.97	3.71	4.62	10.01
Ketone	2-undecanone	11.81	1605	0.25	0.34	0.27	0.76	0.53	n.d.	n.d.	n.d.	0.63	n.d.	0.58	n.d.
Alcohol	2-octen-1-ol	12.11	1621	5.39	20.12	13.56	28.67	28.22	30.12	35.16	22.22	21.00	6.56	n.d.	n.d.
Aldehyde	phenylacetaldehyde	12.59	1646	0.55	n.d.	n.d.	n.d.	n.d.	n.d.	n.d.	n.d.	n.d.	n.d.	n.d.	n.d.
Aldehyde	2,4-nonadienal	13.74	1707	1.53	2.78	0.34	2.58	1.97	2.14	1.81	n.d.	1.15	2.14	2.62	0.93
Aldehyde	2,4 decadienal	14.92	1768	0.55	n.d.	n.d.	n.d.	2.51	2.24	0.8	1.9	2.08	0.94	1.64	1.72
FAME	methyl laurate	15.65	1806	1.33	n.d.	n.d.	2.96	n.d.	n.d.	n.d.	n.d.	n.d.	0.45	n.d.	0.86
Aldehyde	2,4 decadienal (*E,E*)	15.81	1814	0.91	2.39	0.31	n.d.	5.62	4.21	1.87	6.14	3.73	1.81	2.88	3.25
Aldehyde	2-phenyl-2-butenal	18.23	1939	0.53	n.d.	n.d.	0.49	n.d.	n.d.	n.d.	n.d.	n.d.	0.62	n.d.	n.d.
FAME	methyl myristate	19.64	2012	1.46	0.78	0.39	1.37	0.95	0.6	0.81	1.4	0.87	1.98	1.63	1.53
FAME	methyl pentadecanoate	21.58	2116	2.6	1.25	0.68	1.45	0.47	0.61	0.88	2.42	0.78	2.23	1.64	2.84
FAME	methyl palmitate	23.46	2221	11.81	3.92	2.68	8.58	3.27	3.32	4.29	7.07	3.36	8.18	5.33	10.43
Alkane	tetracosane	26.86	2400	n.d.	n.d.	n.d.	n.d.	n.d.	n.d.	n.d.	1.53	n.d.	n.d.	n.d.	n.d.
FAME	methyl oleate	27.36	2440	2.15	n.d.	n.d.	n.d.	n.d.	n.d.	n.d.	n.d.	n.d.	1.58	n.d.	1.62

Class of compound	Compound	Rt (min)	RI	*P. ostreatus*											
				CS4			CS5			CS6			LGAM3002		
				WS	GM	OL	WS	GM	OL	WS	GM	OL	WS	GM	OL
FAME	methyl linoleate	28.15	2503	11.12	1.94	1.44	6.15	2.71	1.73	3.93	7.72	2.34	12.29	2.26	8.77

*Results are averages of three replicates; f.w.: fresh weight; CS: commercial strains; LGAM: Laboratory of General and Agricultural Microbiology (Agricultural University of Athens, Greece); FAME: fatty acid methyl esters; WS, wheat straw; GM, wheat straw with grape marc (1:1 w/w); OL, olive leaves with olive mill wastes (3:1 w/w ratio).

Figure S1. GC/MS chromatogram of volatiles extracted by SPME from *P. eryngii* CS1 mushrooms cultivated on wheat straw. Compounds: 1: toluene; 2: n-hexanal; 3: undecane; 4: heptanal; 5: 3-octanone; 6: 1-octen-3-one; 7: 2,3-octanedione; 8: 2-heptenal; 9: 3-octanol; 10: 2-octenal; 11: 1-octen-3-ol; 12: benzaldehyde; 13: 2,4-nonadienal; 14: methyl pentadecanoate; 15: methyl palmitate; 16: methyl oleate; 17: methyl linoleate

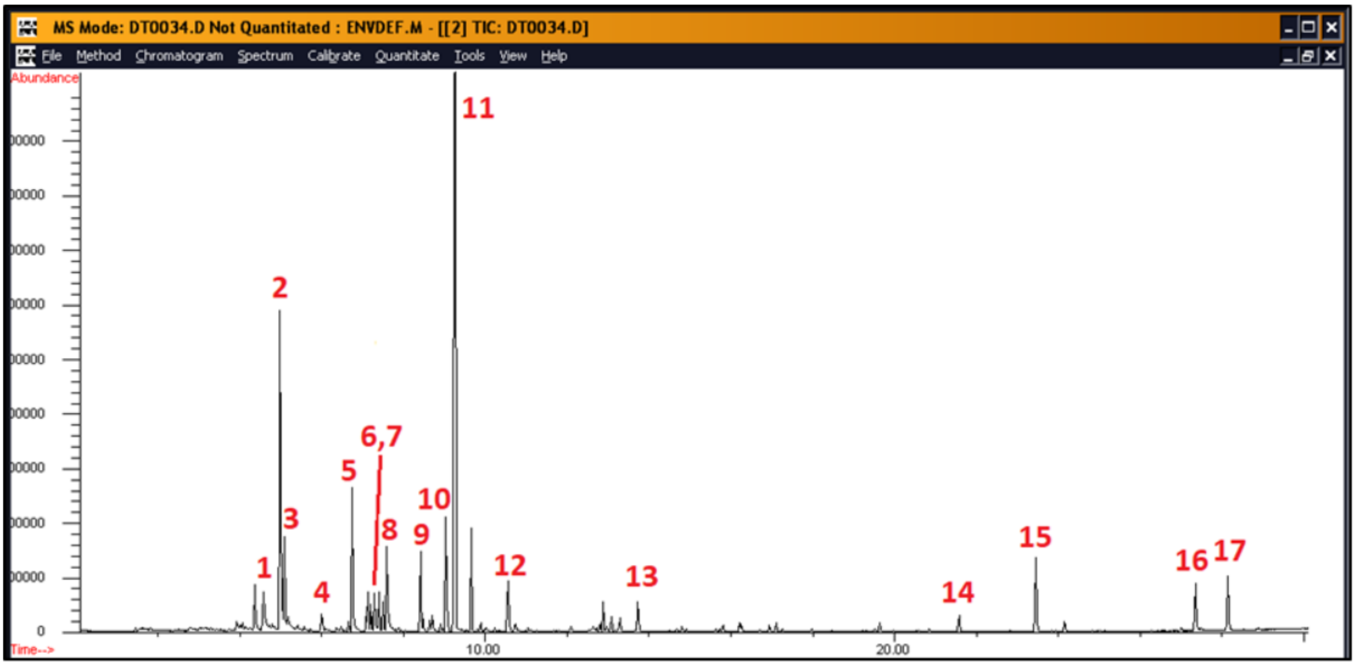

Figure S2. GC/MS chromatogram of volatiles extracted by SPME from *P. ostreatus* CS4 mushrooms cultivated on GM. Compounds: 1: toluene; 2: n-hexanal; 3: 3-octanone; 4: n-octanal; 5: 1-octen-3-one; 6: 2-heptenal; 7: 3-octanol; 8: 2-octenal; 9: 1-octen-3-ol; 10: benzaldehyde; 11: n-octanol; 12: 2-octen-1-ol; 13: methyl palmitate; 14: methyl linoleate.

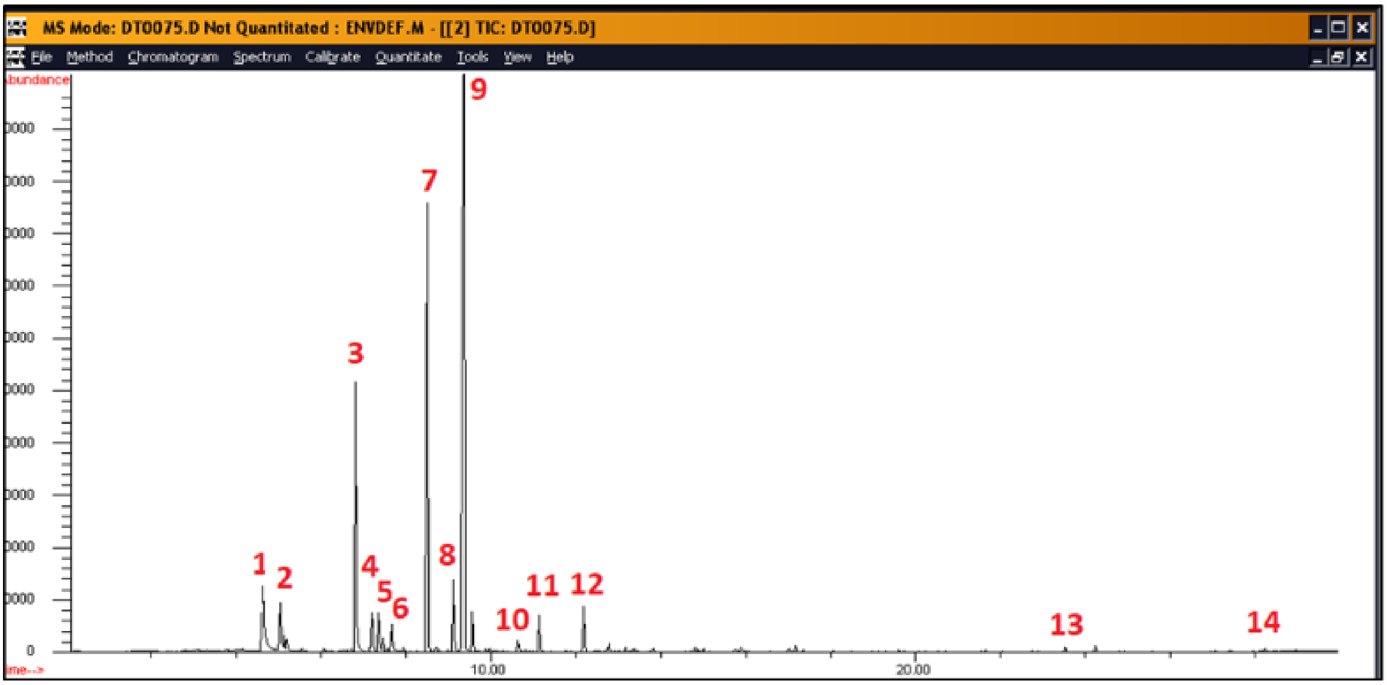

Supplement: Supplementary file 1 [file foods-10-01287-s001.zip › foods-1213612-supplementary.pdf]
